# Acoustic Radiation Force Impulse Elastography for the Non-Invasive Evaluation of Hepatic Fibrosis in Non-Alcoholic Fatty Liver Disease Patients: A Systematic Review & Meta-Analysis

**DOI:** 10.1371/journal.pone.0127782

**Published:** 2015-07-01

**Authors:** Haixia Liu, Jing Fu, Ruixia Hong, Li Liu, Fang Li

**Affiliations:** 1 Department of Ultrasonic Medicine, Chongqing Cancer Institute, No. 181 Han Yu Road, Shapingba District, Chongqing, 400030, China; 2 Department of Internal Medicine, Chongqing Cancer Institute, No. 181 Han Yu Road, Shapingba District, Chongqing, 400030, China; Università degli Studi di Palermo, ITALY

## Abstract

**Background:**

In order to better monitor non-alcoholic fatty liver disease (NAFLD) patients at higher risk for HCC, there is a need for non-invasive diagnostic approaches to screen for the presence of advanced fibrosis in these patients. The aim of this systematic review and meta-analysis will be to evaluate the diagnostic efficacy of ARFI elastography in detecting hepatic fibrosis in NAFLD patients.

**Methods:**

Relevant studies were identified from systematic searches of several major electronic databases (PubMed, EMBASE, and the Cochrane Central Register of Controlled Trials). The primary outcomes were the summary sensitivity, summary specificity, the diagnostic odds ratio, and the summary receiver operating characteristic curve (SROC) of ARFI elastography in detecting significant fibrosis (defined as 4>F≥2) in NAFLD patients. Study quality was assessed using the Quality Assessment of Studies of Diagnostic Accuracy included in Systematic Review (QUADAS-2).

**Results:**

The summary sensitivity and specificity of ARFI in detecting significant fibrosis were 80.2% (95% confidence interval (CI): 0.758–0.842; *p* = 0.0000) and 85.2% (95% CI: 0.808–0.890), *p* = 0.1617), respectively. The pooled diagnostic odds ratio of ARFI in detecting significant fibrosis was 30.13 (95% CI: 12.08–75; chi-squared = 14.59, *p* = 0.0237). The area under the SROC curve (AUC) was 0.898 (standard error (SE): 0.031) with a Q* index of 0.830 (SE: 0.033).

**Conclusions:**

ARFI elastography appears to be modestly accurate in detecting significant fibrosis in NAFLD patients. Future studies in this field should provide head-to-head comparisons of ARFI elastography versus other elastographic imaging modalities in NAFLD patients.

## Introduction

Non-alcoholic fatty liver disease (NAFLD) is characterized by hepatic steatosis in the absence of secondary causes such as high alcohol consumption, medication use, or hereditary factors and has been clinically associated with metabolic disorders such as obesity, diabetes mellitus, and dyslipidemia [1]. NAFLD cases are categorized as either non-alcoholic fatty liver (NAFL; defined as hepatic steatosis with no evidence of hepatocellular injury) or non-alcoholic steatohepatitis (NASH; defined as hepatic steatosis with hepatocellular injury) [1]. Notably, NAFLD patients are at increased risk for developing hepatocellular carcinoma (HCC), but this risk is primarily limited to those patients with advanced fibrosis [1, 2]. Therefore, in order to better monitor NAFLD patients at higher risk for HCC, there is a need for non-invasive diagnostic approaches to screen for the presence of advanced fibrosis in these patients.

To this end, several non-invasive methods to identify advanced fibrosis in NAFLD patients have been investigated, including the NAFLD Fibrosis Score (based on age, BMI, hyperglycemia, platelet count, albumin, and aspartate aminotransferase/alanine aminotransferase [AST/ALT] ratio), the Enhanced Liver Fibrosis (ELF) panel (based on plasma levels of hyaluronic acid, TIMP-1, and PIIINP), plasma levels of cytokeratin-18 (CK18) fragments, and liver stiffness measurement by elastographic imaging methods (e.g., transient elastography, real-time transient elastography (RTE), acoustic radiation force impulse (ARFI) elastography, and magnetic resonance elastography (MRE)) [1, 3, 4].

Although the NAFLD Fibrosis Score is based on readily-available variables that are typically collected as part of the routine check-up for NAFLD patients, elastographic imaging modalities for liver stiffness have an inherent advantage in terms of immediacy over more complex biomarker assays such as ELF and CK-18, as elastography does not require the time-consuming blood sampling and laboratory processing. The most conventional and well-studied of these imaging methods is transient elastography, which is marketed as FibroScan by Echosens (Paris, France) [5]. Although transient elastography can detect advanced fibrosis, it displays reduced applicability in NAFLD patients, a phenomenon which is still not completely ameliorated by the new XL probe [6]. Moreover, RTE is not highly accurate in staging liver fibrosis, and the relatively novel MRE remains understudied in NAFLD populations [4, 7].

In contrast, acoustic radiation force impulse (ARFI) elastography–a technology marketed by Siemens which integrates elastography and conventional B-mode ultrasonography—displays a higher rate of reliable measurements and a similar diagnostic efficacy to transient elastography in detecting significant fibrosis according to a recent meta-analysis by Bota et al. [8]. However, Bota et al.’s work did not specifically assess the diagnostic efficacy of ARFI elastography in detecting hepatic fibrosis in NAFLD patients. Therefore, the aim of this systematic review and meta-analysis will be to evaluate the diagnostic efficacy of ARFI elastography in detecting hepatic fibrosis in NAFLD patients.

## Materials and Methods

### Search Strategy

We performed a systematic review of the current literature according to the Preferred Reporting Items for Systematic Reviews and Meta-Analyses (PRISMA) guidelines (S1 Table) [9]. Relevant studies were identified from systematic searches of several major electronic databases (PubMed, EMBASE, and the Cochrane Central Register of Controlled Trials) up to July 2014 with different combinations of the following key words: (ARFI OR "acoustic radiation force impulse") AND (liver or hepatic) AND (fatty OR NAFLD OR NASH OR steatohepatitis) AND fibrosis. Additional relevant articles were obtained by scanning reference lists of the articles identified in the initial searches. An English language restriction was imposed.

### Study Selection and Data Extraction

Two authors independently screened the titles and abstracts for eligibility and the full texts for final inclusion. Those studies not evaluating ARFI elastography in NAFLD patients and studies evaluating children were summarily excluded. Animal studies, reviews, case reports, supplements, abstracts, and posters were also excluded.

The following data were extracted from each study: first author’s name, country, publication year, number of study participants undergoing ARFI elastography (n), histopathological liver diagnosis (NAFL or NASH), technical failures of ARFI elastography (if any), histological scoring system used for fibrosis evaluation, physical dimensions of liver biopsy samples, Spearman's correlation coefficient for ARFI and fibrosis as well as ARFI cut-off values, sensitivity, specificity, and the area under the receiver operating characteristic curves (AUC) for the different stages of fibrosis (F = 1–4). When reported, we also extracted the number of true positive, true negative, false positive, and false negative results of ARFI for significant fibrosis (defined as 4>F≥2).

### Methodological Quality

The quality of the studies was assessed using the Quality Assessment of Studies of Diagnostic Accuracy included in Systematic Review (QUADAS-2).

### Outcomes & Statistical Analysis

The primary outcomes were the summary sensitivity, summary specificity, the diagnostic odds ratio, and the summary receiver operating characteristic curve (SROC) of ARFI elastography in detecting significant fibrosis (defined as 4>F≥2) in NAFLD patients. Meta-DiSc v1.4 (Clinical Biostatistics Unit, Ramo y Cajal Hospital, Madrid, Spain) was used to perform all statistical analyses. A random-effects model was used to compute the summary estimates. A *p*-value of less than 0.05 was deemed statistically significant for all analyses.

## Results

**Fig 1:**
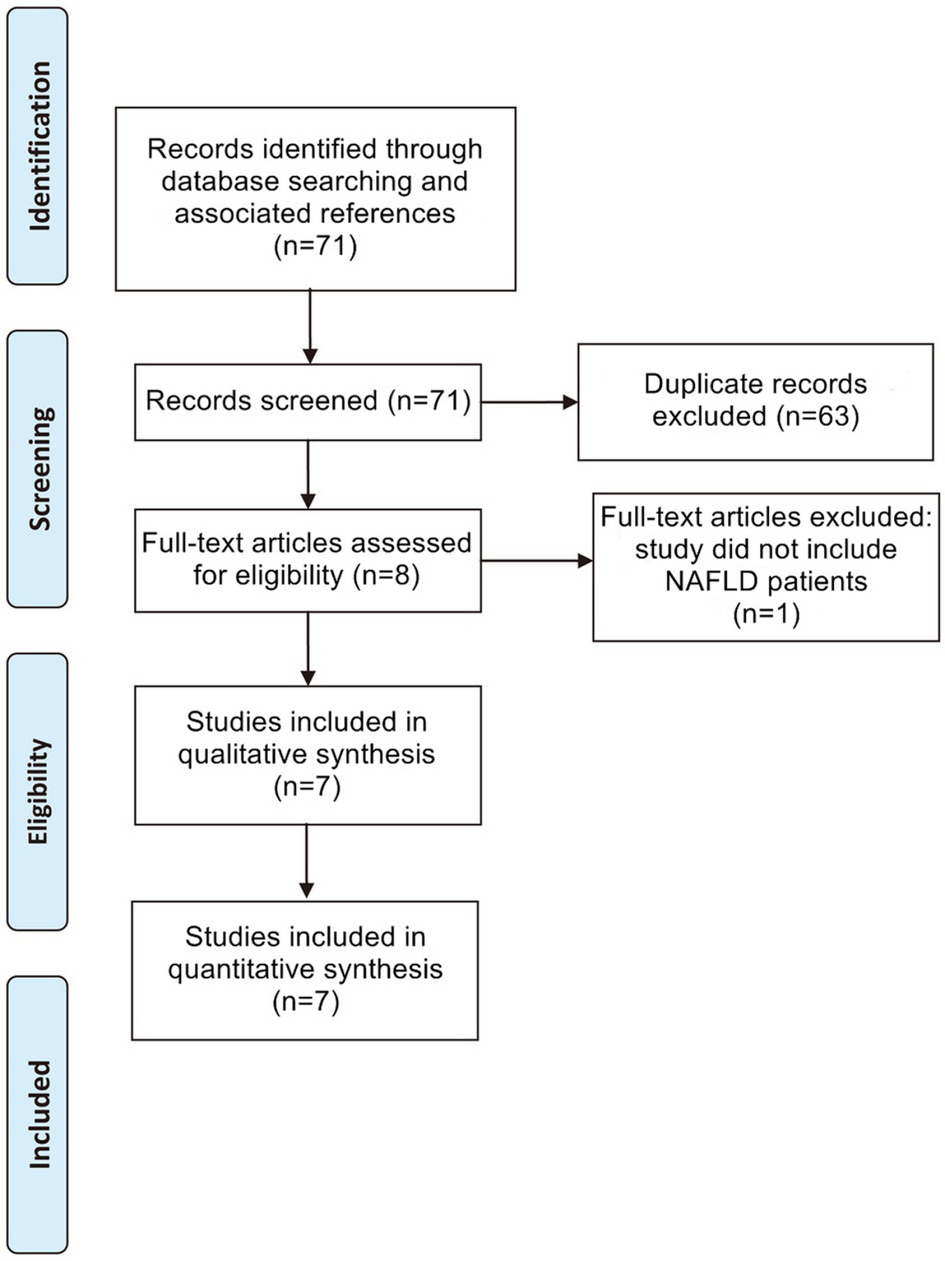
Flowchart of Study Selection. Flowchart depicting the study selection process for this systematic review and meta-analysis.

The flowchart detailing the study selection process is provided in Fig 1. After application of all inclusion and exclusion criteria, seven studies consisting of a total of 723 patients that underwent ARFI elastography were finally included in this meta-analysis [10–16]. The characteristics of these seven studies are detailed in Table 1, and the quality assessment of the studies by QUADAS-2 is shown in Table 2.

**Table 1:** Characteristics of Included Studies.

Study	Country	n	Dx	Tech fail	Histological score used	Biopsy	R	Cut-off values (m/s)	Sn	Sp	AUC
Braticevici 2013	Romania	64	NAFLD	NR	Metavir	At least 2 cm length	0.843	F≥1:1.105	76.7	71.4	
								F≥2:1.165	84.8	90.3	0.944
								F≥3:1.480	86.4	95.2	0.982
								F = 4:1.635	91.7	92.3	0.984
Cassinotto 2013	France	321	NASH*	NR	Metavir	Median length: 25 mm	NR	F≥2:1.38	71	78	1.38
								F≥3:1.51	75	80	1.57
								F = 4:1.61	82	74	1.61
Friedrich-Rust 2011	Germany	57	NAFLD or NASH	NR	Metavir	Mean length: 22.9 ± 9.4 mm; median length (range): 20 mm (10–48)	NR	F≥2:1.37	97	67	0.84
								F≥3:1.45	76	68	0.91
								F = 4:1.75	74	67	0.91
Guzmán-Aroca 2012	Spain	32	NAFLD	NR	Brunt	-	NR	1.3	85	83.3	
Osaki 2010	Japan	23	NASH	NR	Brunt	-	NR	F≥1:1.34			
								F≥2:1.79			
								F≥3:2.20	100	75	0.942
								F = 4:2.90			
Palmeri 2011	USA	172	NAFLD	NR	Metavir	-	0.22		90	90	0.9
Yoneda 2010	Japan	54	NAFLD	NR	Brunt	At least 2 cm length	NR	F≥3:1.77	100	91	0.93
								F = 4:1.9	100	96	0.97

Abbreviations: Dx, diagnosis; Tech fail, technical failures; R, Spearman’s correlation coefficient between ARFI and fibrosis; Sn, sensitivity; Sp, specificity; AUC, area under the receiver operating characteristic (ROC) curve; NASH, xxxx; NAFLD, xxxxx; NR, not reported

*Only the subpopulation of NASH patients were included.

**Table 2:** Quality Assessment of Studies of Diagnostic Accuracy included in Systematic Review (QUADAS-2).

Study	Risk of bias				Applicability concerns		
	Patient selection	Index test	Reference standard	Flow and timing	Patient selection	Index test	Reference standard
Braticevici 2013	H	L	H	U	U	U	U
Cassinotto 2013	L	L	H	H	U	U	U
Friedrich-Rust 2011	H	L	H	U	U	L	U
Guzmán-Aroca 2012	H	U	H	U	U	L	U
Osaki 2010	H	L	H	U	L	U	U
Palmeri 2011	L	L	L	U	U	L	U
Yoneda 2010	H	H	H	U	L	H	U

Abbreviations: H, high; L, low; U, unclear

**Fig 2:**
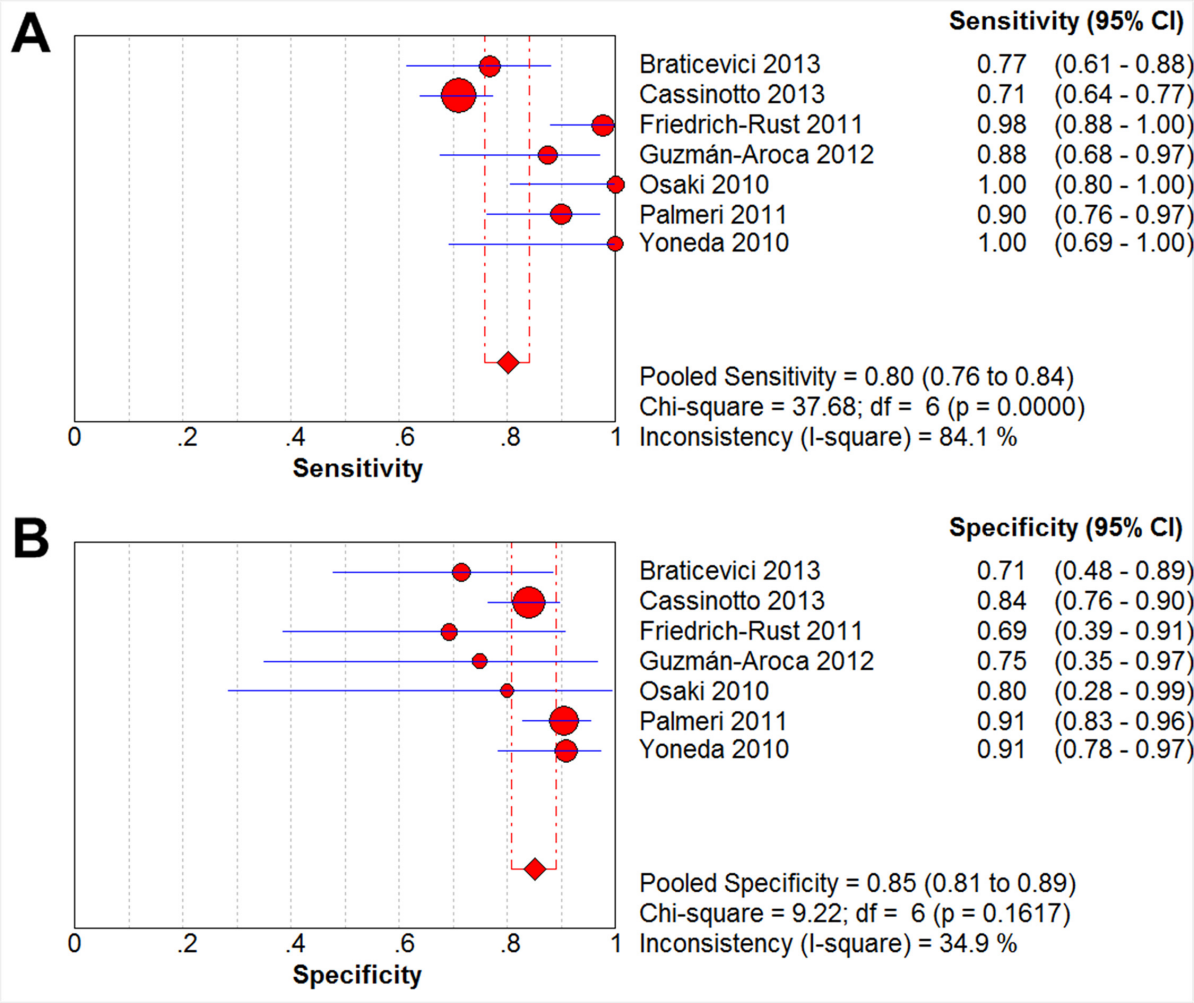
Forest Plots of Sensitivity & Specificity. The red circles and the horizontal blue lines represent the point estimates and 95% confidence intervals (CIs) of the included studies, respectively. The weight of each study is depicted by the size of its respective circle. The center of the red diamond represents the pooled estimate, and the red dotted lines represent the 95% CI of the pooled estimate.

Here, we analyzed the diagnostic efficacy of ARFI elastography in detecting significant fibrosis (defined as 4>F≥2) in NAFLD patients by pooling the sensitivities, specificities, diagnostic odds ratios, and receiver operating characteristic (ROC) curves from the seven included studies. A high sensitivity shows efficacy at ruling out a disease, while a high specificity shows efficacy in confirming a disease [17]. From our meta-analysis, the summary sensitivity of ARFI in detecting significant fibrosis was found to be 80.2% (95% CI: 0.758–0.842; *p* = 0.0000; Fig 2A), and the summary specificity of ARFI in detecting significant fibrosis was determined to be 85.2% (95% CI: 0.808–0.890), *p* = 0.1617; Fig 2B).

**Fig 3:**
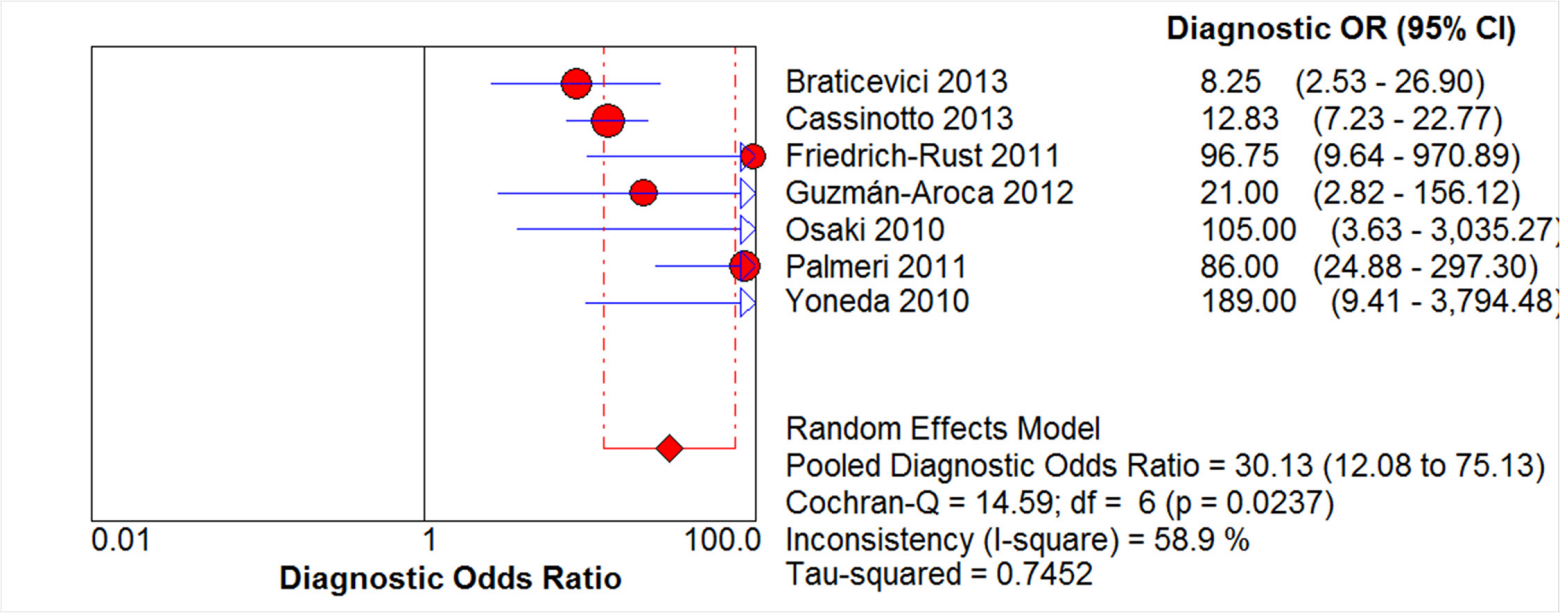
Forest Plot of Diagnostic Odds Ratio. The red circles and the horizontal blue lines represent the point estimates and 95% confidence interval (CIs) of the included studies, respectively. The weight of each study is depicted by the size of its respective circle. The center of the red diamond represents the pooled estimate, and the red dotted lines represent the 95% CI of the pooled estimate.

The pooled diagnostic odds ratio–which describes the odds of a positive test result in a diseased participant as compared to the odds of positive test result in a non-diseased participant—is a more robust measure than pooled sensitivity and specificity values, as its value stays relatively constant regardless of the diagnostic threshold applied [17]. The diagnostic odds ratio of ARFI in detecting significant fibrosis was found to be 30.13 (95% CI: 12.08–75; chi-squared = 14.59, *p* = 0.0237; Fig 3).

**Fig 4:**
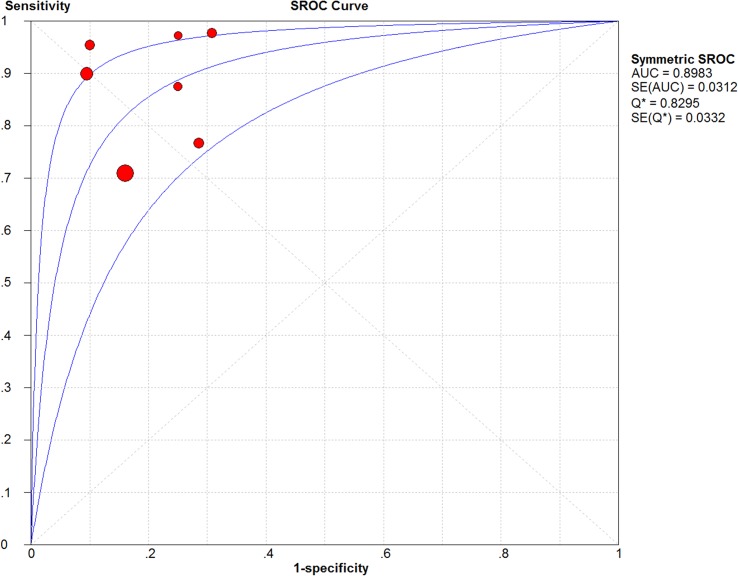
Summary Receiving Operating Characteristic Curve (SROC) Plot. The SROC and its 95% confidence interval (CI) boundaries are represented by the three blue lines. Each of the included studies is represented by a red circle with the weight of each study depicted by the size of its respective circle. The area under the curve (AUC) was calculated by numerical integration of the SROC.

Heterogeneity is relatively commonplace in diagnostic meta-analyses and may result from either threshold effects (i.e., the use of different cut-offs for a positive test result), inherent differences in the diagnostic testing methods used, or differences in study characteristics [17]. Here, the SROC plane did not display a classic “shoulder-arm” shape, revealing no significant threshold effect (Fig 4). The area under the SROC curve (AUC)—which displays the trade-off between sensitivity and specificity with a value of 1.0 indicating ideal discriminatory ability [18]—was found to be 0.898 (SE: 0.031). The Q* index—defined by the point where sensitivity equals specificity on the SROC curve with a value of 1.0 indicating 100% accuracy [18]—was determined to be 0.830 (SE: 0.033).

## Discussion

As liver biopsy is not recommended in NAFLD patients due to its risk of complications and conventional B-mode imaging can be inaccurate in NAFLD patients, ARFI elastography is a promising imaging technology that astutely combines elastography and conventional B-mode ultrasonography to measure liver stiffness, a phenomenon which correlates well with liver fibrosis [19, 20]. In this systematic review and meta-analysis, we found that ARFI elastography was modestly accurate (i.e., summary sensitivity: 80.2%; summary specificity: 85.2%; diagnostic odds ratio: 30.13; AUC: 0.898; and Q* index: 0.830) in detecting significant fibrosis (defined as 4>F≥2) in NAFLD patients.

Specifically, ARFI elastography involves targeting a region of interest (5 mm × 10 mm) by B-mode imaging; then, the ARFI ultrasound probe is used to produce short pulses (262 ms at a frequency of 2.67 MHz) that generate shear waves that are tracked using ultrasound, thereby obtaining a quantifiable output in terms of shear wave speed (SWV, measured in m/s) [19]. The vast majority of studies have shown that the SWV increases with the degree of fibrosis observed by histopathology [21]. Thus, as opposed to conventional elastographic methods, ARFI elastography provides both a qualitative measure of displacement as well as a quantitative measure of SWV, which provides an advantage in assessing hepatic fibrosis [19].

That being said, SWV measurement by ARFI elastography can be complicated by the steatosis and hepatic inflammation present in the NAFLD liver [4, 21]. Although Palmeri et al. found no relationship between SWV values and hepatocyte ballooning or inflammation [15], Braticevici et al. reported significant SWV decreases in proportion to increasing steatosis severity [4, 10]. Moreover, Yoneda et al. reported that SWV values significantly varied between NAFLD patients with different hepatic inflammatory activity levels [4, 16]. Therefore, based on the foregoing evidence, steatosis appears to decrease SWV values, while hepatic inflammation appears to increase SWV values in NAFLD patients undergoing ARFI elastography [4]. Although here we found that ARFI elastography was modestly accurate in detecting significant fibrosis in NAFLD patients, we could not specifically assess the effects of steatosis and hepatic inflammation upon ARFI elastography’s accuracy because this type of data is not typically reported in ARFI elastography studies. Therefore, future trials of ARFI elastography in NAFLD patients should histopathologically assess hepatic inflammation and steatosis in order to better determine their effects upon SWV values.

There are several limitations to this study. First, because ARFI elastography is a relatively new technology that has not been extensively investigated in NAFLD patients, only seven studies consisting of a total of 723 patients that underwent ARFI elastography were finally included in this meta-analysis. Second, due to the paucity of comparative trials, this study could not provide a head-to-head comparison of ARFI elastography versus transient elastography, RTE, MRE, or other more novel elastographic imaging technologies (e.g., two-dimensional shear wave elastography, strain elastography) in NAFLD patients [4, 21]. Third, as the included studies failed to report on observed technical failures of ARFI elastography (if any), we are uncertain as to whether any technical failures did occur, and if so, what the nature of such technical failures were. Fourth, previous research has reported losses in diagnostic accuracy in obese patients and individuals with chronic hepatitis B [13, 21, 22]; however, we were unable to analyze the effects of elevated BMI or chronic hepatitis B infection status upon the diagnostic efficacy of ARFI elastography here. Fifth, since the sole aim of this study was to assess the use of ARFI elastography in detecting hepatic fibrosis in NAFLD patients, we did not correlate ARFI scores with measures of steatosis. A recent 2014 study found that ARFI scores did not significantly correlate with either steatosis grade or NAFLD score, revealing that ARFI elastography may not be suited for short-term monitoring of NAFLD patients [23].

In conclusion, ARFI elastography appears to be modestly accurate in detecting significant fibrosis in NAFLD patients. Future studies in this field should provide head-to-head comparisons of ARFI elastography versus other elastographic imaging modalities (e.g., transient elastography, RTE, and MRE) in NAFLD patients.

## Supporting Information

S1 Table PRISMA Checklist. (DOC)
